# A Case of Clairvoyance

**Published:** 1888-11

**Authors:** Kathie Moore


### A CASE OF CLAIRVOYANCE.

Before relating the curious occurrence which came under my observation a short time ago, and which appears to me one of the most inexplicable of mental phenomena, I wish it to be clearly understood that I am a most thorough skeptic in the so-called clairvoyancy, supernatural dreams and spiritualism of the day. We see so many remarkable co-incidences happening among our friends that we almost cease to be surprised at them ; but even when considered only as co-incidences, these remarkable happenings are worthy of a place in the list of unexplained mysteries.

While visiting Pasadena, Cal., a few months ago, I made the acquaintance of an intelligent young girl who, partly because of a peculiar affliction, and partly from force of circumstance, lived a very secluded life. She and her mother had come from the East a short time before, and were then living about two miles from the town, on a lonely, rented estate. When I first made their acquaintance I found that they had not a single friend in the place, and were, of course, very lonely. We soon became fast friends, and as I had nothing else to do but to enjoy myself, it came to pass that I spent all my afternoons with my young friend. As she was unable to leave her home the visiting was entirely on my side. One evening as I was about to say good-night to her she said : "I had a dream about you that troubled me a little last hight. I dreamed that you stayed away for a whole week, and as we had no means of sending for you, and you did not send to us, we were very much worried and feared you were ill."

I laughed and remarked that I had never been ill in my life, and had no idea of becoming so ; nevertheless my young friend seemed troubled, and replied : "Of course I don't believe in dreams, but if you should feel yourself getting ill, won't you come and stay with us until you recover ? You would be so uncomfortable at the boarding house, and mamma and I would be so lonely without you."

This was such a genuinely kind invltation that I responded to it in the spirit in which it was given, and repled that I would do as she wished. I was suffering from a slight cough at the time, and as the doctor had given me a counter-irritant to use, I applied it that night for the first time. I used my irritant so generously that the next morning saw me converted into an enormous blister, and for five days I was confined to the house. The seventh day I secured a driver and went to see my young friend. The words she greeted me with, as she looked anxiously into my pale face, were :

"I *dreamed* you would be sick for a week."

A few days afterward I drove to her door and invited her to go with me to Sierra Madra, a few miles distant. Imagine my surprise when she exclaimed, in a disappointed tone :

"Sierra Madra ? I thought it was to be Los Angeles !"

Upon inquiry I found that she had dreamed that I had come to take her for a drive, though she had not dreamed that our destination was to be Los Angeles. Something had given her the impression that it was so. Another time when I was anxiously awaiting a telegram from Philadelphia, I remarked to her that I was going to town to see whether it had arrived, when she said quickly :

"You need not go, it isn't there."

"Why," I asked, "did you dream about it ?"

She flushed, hesitated, and then replied that what she called *dreams* were not really dreams, that is, they did not come to her in her sleeping hours, but while she was awake ; she merely called them dreams because she had no better name for them. In the meantime she had had a number of these "dreams," all quite as remarkable, though more trifling than those here related,and as I was becoming interested in these phenomenal visions, I pushed my inquiries, and found that the dreams came only in relation to myself. She never had such visions in connection with anyone else. She seemed very much surprised at her sudden ability to fortell events, and very innocently said that "the dreams just come to me like a thought." Several times I tried to bring on one of these prophetic states by asking her questions, but it was evidently a matter of inspiration with her—she could not answer a question relating to the future, and laughingly declared : "I don't know," in answer to all such queries. But about that telegram. We drove to the telegraph office, and found, as she had said, that it had not come. When I told her so, she remarked, in the quick way in which she always told the "dreams," as though a thought had just struck her :

"It won't come before the latter part of next week." And she was right—indeed the *telegram* did not come at all, but the following Thursday brought me a letter instead.

There were, as I have stated before, a number of similar incidents, all of which are too trifling to relate, but all quite as remarkable, in a psychological sense, as the ones I have described. About this time it became necessary for me to make a change in my residence, and as the question of "where" was one of great importance, though, at the same time, entirely one of choice, I was for several weeks quite uncertain and a little troubled about making a decision. Thinking that my little clairvoyant, as I had begun to call her, might help me, I put the question to her a number of times :

"Where shall I go? Is Beaumont or Denver the place for me?" to which she invariably replied : "I can't tell."

"I wish you would try and find out," I urged. "See whether you can't 'dream' about it."

"I have tried, but it does no good. I never can "dream" when I try," she answered.

A short time after this I decided to go to Denver. When I told her so, she answered, in her quick way :

"Denver isn't the place for you. You won't do well there."

However, I went there, and circumstances proved her to be right. Financially, Denver was a failure. While there I kept up a brisk correspondence with my young friend, and in nearly every letter she gave me some proof of her clairvoyance, or whatever her strange power was. For instance, a week before Christmas she wrote : "Something important is going to happen to you about Christmas time;" and a few days after the important thing happened, she wrote me in detail a lengthy "dream," describing, in a sort of allegorical way, the very thing that had occurred, and which was of great moment to me. All the time of my absence I had represented myself as in the best of health, and yet in one of her letters she said :

"Don't you think you had better leave Denver as soon as possible? I am afraid you will have a return of your old lung trouble, or are you suffering from it already?" To which I replied that I *was* suffering from it, and contemplated a speedy change.

I know that the young lady could not have ascertained these things of which she professed to "dream." She had no acquaintances in Denver, and none at all who were acquainted with me. Indeed, many of her revelations were of such a nature that no one could possibly have known that such things were about to take place—that is, no one in the ordinary run of affairs could have foreseen them. What appears most strange to me is that her clairvoyance, or what you will—I call it that for want of a better name—extended to no one but myself, and was as great a mystery to her as to me.

It is now several months since I have seen her, and though we still exchange letters, her prephetic dreams are not so numerous as before. She explains this by saying that, as we are so widely separated by time and space, our interest in each other is not so intense, and that naturally her mind is not so occupied with thoughts of me. Nevertheless, she still treats me occasionally to some of her "dreams," which are always as true as those of which I have spoken.—KATHIE MOORE in *The Phrenological Journal.*

